# Human health risk assessment of heavy metals in vegetables of Bangladesh

**DOI:** 10.1038/s41598-024-65734-6

**Published:** 2024-07-06

**Authors:** Akibul Islam Chowdhury, Lincon Chandra Shill, M. Maruf Raihan, Rumana Rashid, Md. Nizamul Hoque Bhuiyan, Sompa Reza, Mohammad Rahanur Alam

**Affiliations:** 1 Department of Food Technology and Nutrition Science, Noakhali Science and Technology University, Noakhali, 3814 Bangladesh; 2 Department of Nutrition and Food Engineering, Daffodil International University, Savar, Dhaka, Bangladesh; 3 Department of Public Health Nutrition, Primeasia University, Dhaka, Bangladesh; 4 Institute of Nutrition and Food Science, University of Dhaka, Dhaka, Bangladesh; 5 Department of Nutrition, University of Tennessee, Knoxville, TN USA

**Keywords:** Heavy metal, Vegetables, Carcinogenic, Non-carcinogenic, Atomic absorption spectrometry, Plant sciences, Ecology, Environmental sciences, Natural hazards, Risk factors

## Abstract

This study aims to evaluate the heavy metal concentration in fifteen species of vegetables as well as associated health risk. Atomic absorption spectrometry is used to assess heavy metals. The mean concentrations of Pb, Cd, Cr, Ni and Fe in vegetables were 4.78, 0.713, 9.266, 0.083, 5.06 mg/kg/fw exceeding the reference value of FAO/WHO indicating unsafe to consumption. Based on principal component analysis, the Pb, Cr, Ni and Fe are from same sources. Health risk was estimated in terms of estimated daily intake (EDI), target hazard quotient, hazard index (HI) and cancer risk (CR). The EDI values of metals except Cr were found to be lower than maximum tolerable daily intake (MTDI). The total THQs of metals were > 1 indicating non-carcinogenic health risk. The individual HI values for vegetables except potato (0.831) and total HI values were found to be > 1 (94.747). The TCR of Pb, Cd and Cr were > 1.0E−04 which indicating carcinogenic risk. Fruit and pod vegetables contribute much in carcinogenic risk for Pb and Cr whereas fruit, root and stems vegetables for Cd. The study revealed potential human health risk associated with the consumption of different types of vegetables in Bangladeshi adult population that might assist the regulatory bodies to develop new strategies to minimize the risk to human.

## Introduction

With the increasing risk of human health through consumption of contaminated foods by heavy metals, pesticides, chemical fertilizers and toxins, the concern regarding food safety issues is attracted to many environmental scientist^1,2^. Consumption of different types of vegetables are crucial for human health as they supply fiber and different types of vitamins and minerals required for growth and tackling different associated deficiencies^3^. So, safety of vegetables for human from pollution are optimal^4^. Plants especially vegetables contain both essential and optimal metals. Contamination such as toxic metals in agricultural production may come from climate, environmental pollution, wasted water, industrial waste, soil, use of chemical fertilizers and pesticides^5–7^.

Metals found in vegetables have both positive and negative roles in human health, however, intake of toxic metals from vegetables have adverse health effects. Heavy metals such as Cd, Pb, Cu, Cr and As are considered most toxic metals by US Environment Protection Agency (USEPA)^8,9^. Bioaccumulation of these heavy metals in vegetables may cause carcinogenic or mutagenic effect in human body prior to ingestion. Pb and Cd are most harmful elements for human health which cause breathing problems, cardiovascular disease, kidney disease, neurological problems and bone disease etc.^10,11^. Chromium (Cr) is commonly found in soil and rock which effects the biological process in various plants and vegetables^12^. Ingestion of Cr contaminated vegetables may cause DNA damage, carcinogenic and mutagenic effects^11^.

In Bangladesh, vegetables are one kind of main foodstuffs consumed by people on regular basis. Consumption of vegetables by Bangladeshi people ranges from 70 to 191 g/day depending on types of vegetables^13^. So, assessment of heavy metals consumed by Bangladeshi population is necessary to evaluate the health risks^7,14^. In Bangladesh, different types of vegetables are grown throughout the year. Evaluation of heavy metals in vegetables are assessed in many previous studies^6,7,14,15^ but on a specific region basis data are very limited and no study yet compare the differences of heavy metals in different types of vegetables.

Noakhali is a coastal region of Bangladesh where different types of industries are established. Heavy metal pollution in coastal region is very common due to discharge of heavy metals in coastal water by several anthropogenic activities such as food processing, pharmaceuticals, paper industry etc.^16,17^. This have significant consequences on environment due to bio-magnification in food chains^18^. However, there is no specific data of heavy metals contamination in vegetables grown in the area of Noakhali. As there is lack of information about heavy metal contents in most common consumed vegetables in Noakhali, Bangladesh as well as differences of metal concentration among vegetable types, the study aims to represent metals concentration in vegetables and potential carcinogenic and non-carcinogenic health risk for these vegetables’ consumption. The study also evaluates the sources and differences of heavy metals in the vegetables.

## Methodology

### Sampling and Study area

**Figure 1:**
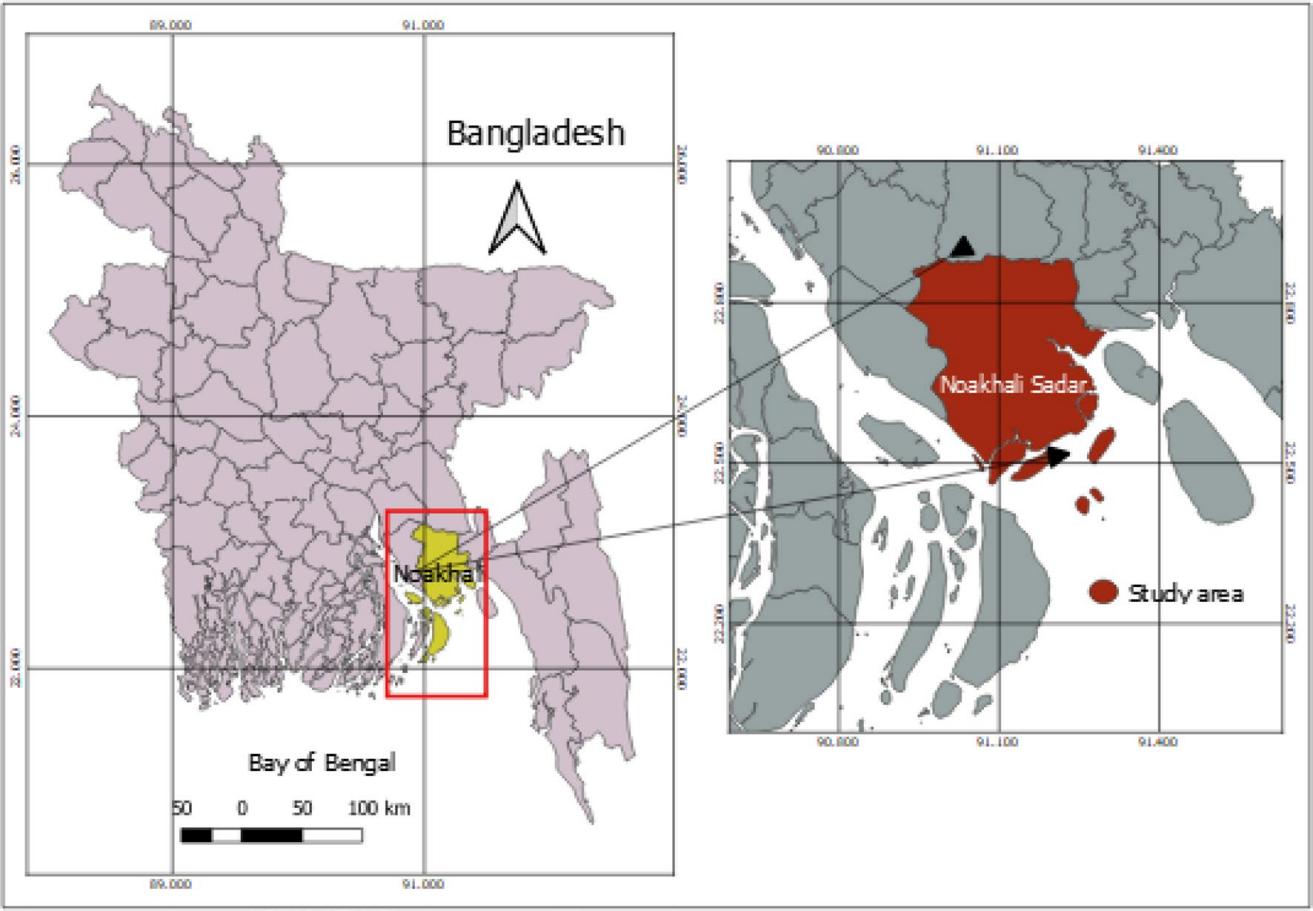
Sampling site of the study, Noakhali, Bangladesh prepared by QGIS software (version 3.10.2) (https://www.qgis.org/en/site/).

The study was carried out in Noakhali district (N22°49′28.7″, E91°6′6.24″) of Bangladesh during the year of 2022. Fifteen different sample species of vegetables were collected from three different local markets located in Noakhali district. The sampling sites were shown in Fig. 1 which was created by QGIS software (version 3.10.2). For each species, 3 replicates vegetable sample were collected from each market. In total, 135 samples were collected from three local markets. All samples were washed and stored in fresh polybag and brought to laboratory for analysis.

### Wet digestion

In the sample preparation, all samples were cleaned with deionized water and chopped with stainless steel blade. Then, all samples were dried in oven at 100^0^C to remove all moisture and grinded with the help of a mortar. Wet digestion method was used with a slight modification of the method by Akinyele et al.^19^. To digest the samples, acid mixture was used (69% concentrated HNO_3_:95–97% concentrated H_2_SO_4_ = 1:4). According to digestion, 0.5 g of each homogenous dried sample was placed in digestion tube and added 5 ml of acid mixture into it. Then the mixture was heated from 130–170 °C for an hour until transparent solution was observed. After cooling, 2 ml of H_2_O_2_ (30% concentrated) was added and heated until clear solution was found. Then the clear solution was filtered using Whatman 102 filter paper and then diluted to 250 ml of deionized water. Then, the samples were collected in falcon tube and stored for analysis by atomic absorption spectrometry.

### Atomic absorption spectrometry (AAS)

The heavy metal determination was carried out using a Perkin-Elmer novAA 900H atomic absorption spectrometer^20^. To detect each metal, a single beam hollow cathode lamp of chromium, cadmium, lead, iron, and nickel were used at specific wavelengths. Both graphite furnace (Cr, Cd, Pb, Ni) and flame (Fe) techniques were used.

### Quality control

To ensure the quality control, blank sample was used in every sample analysis. The reliability of each method was evaluated by linearity, range and recovery. The procedure of validation of result were summarized in Supplementary Tables 1 and 2. The percentage of recovery (% R) was ranged from 97.15 to 101.55% and the linearity was ranged from 0.995 to 0.998. Effective aseptic technique was followed to prevent contamination in samples and all glass materials were rinsed with 1% nitric acid.

### Estimated daily intake (EDI) of heavy metals

EDI was measured in mg/kg-weight by following formulae^21^.

$${\text{EDI }} = \frac{MC \times IR}{{BW}}$$

Where MC is the metal concentration in vegetables (mg/kg fw), IR (g/day/person) is the ingestion rate of vegetables adopted from household income and expenditure survey (HIES)^13^ for an adult individual of 60 kg (adult) body weight^22^.

### Non-carcinogenic risk

The non-carcinogenic risk of heavy metals due to consuming vegetables were estimated in terms of target hazard quotient (THQ), total target hazard quotient (TTHQ), and hazard index (HI).

#### THQ

THQ was calculated as per USEPA Region III Risk-based Concentration Table ^23^ and Wang et al.^24^. The equation used for estimating THQ was;

$$TH{\text{Q}} = { }\frac{EF \times ED \times FIR \times CM}{{BW \times {\text{A}}T \times RfD}} \times 10^{ - 3}$$

where EF is the exposure frequency (365 days/year); ED is the exposure duration (70 years for non-cancer risk in this study), as used by^25^; FIR is the food ingestion rate (g/person/day); CM is the heavy metal concentration in vegetable (mg/kg); BW is the average body weight (bw) (adult: 60 kg); AT is the average exposure time for non-carcinogens (EF × ED) (365 days/year for 70 years; i.e. AT = 25,550 days); RfD is the oral reference dose of the metal. RfD values of Cd, Cr, Ni, Pb and Fe are 0.001, 1.5, 0.02, 0.0035 and 0.667 (mg/kg/day), respectively^26^.

If the THQ < 1, the exposed consumers are unlikely to experience any adverse health risk, while if the THQ ≥ 1, there is a potential health risk^24^, and associated interventions and protecting initiatives are required to be taken.

#### TTHQ

TTHQ for individual from THQs is expressed as the sum of the hazard quotients^25^.

$$TTHQ = TH{\text{Q}}\left( {{\text{Cr}}} \right) + TH{\text{Q }}\left( {{\text{Pb}}} \right) + {\text{THQ}}\left( {{\text{Cd}}} \right) + {\text{THQ }}\left( {{\text{Ni}}} \right) + {\text{THQ}}\left( {{\text{Fe}}} \right)$$

where TTHQ < 1 is safe, TTHQ > 1 is hazardous, and THQ (Cr) is the target hazard quotient for Cr intake.

#### Hazard risk (HI)

HI is assessed to estimate overall potential for non-carcinogenic health risk from consuming more than one metal.

$${\text{HI }} = {\text{ TTHQ }}\left( {{\text{food}}_{{1}} } \right) \, + {\text{ TTHQ }}\left( {{\text{food}}_{{2}} } \right) \, + \cdots + {\text{ TTHQ }}\left( {{\text{food}}_{{{15}}} } \right)$$

### Carcinogenic risk assessment

#### Target cancer risk

The method to estimate TCR is also provided in USEPA Region III Risk-Based Concentration Table^25^. The model for estimating TCR was shown as follows (4):

$$TCR = EDI \times CPSo$$

where EDI is the estimated daily intake, CPSo is the carcinogenic potency slope for oral route of 0.0085 (mg/kg-day)^−1^ for Pb, 15 (mg/kg-day)^−1^ for Cd, and 0.42(mg/kg-day)^−1^ for Cr^27^. In general, CR value lower than 1.0E−06 is considered to be negligible, above 1.0E−04 is considered unacceptable, and lying between 1.0E−06 and 1.0E−04 is considered an acceptable range ^28,29^. Data required for estimating EDI, THQ and CR are summarized in Supplementary Table 3.

#### Ethical consideration

The study was classified as exempt according to the institutional ethics committee of the Noakhali Science and Technology University. All methods were performed in accordance with the relevant guidelines and regulations.

### Statistical analysis

All statistical analyses were performed with SPSS 23.0 Inc., Chicago, IL, USA for Windows. Data were presented as mean and standard deviation (SD) and were subjected to one-way analysis of variance (ANOVA) to assess whether heavy metals varied significantly between vegetables (*p* < 0.05). To check the similarities and differences of heavy metals distribution, principal component analysis (PCA) and cluster analysis (CA) were performed. The PCA analysis was done using Varimax normalized rotation method to maximize the sum of the variance of the factor coefficient and CA was performed using Ward’s method.

## Results and discussion

### Concentration of heavy metals in commonly consumed vegetables

Heavy metal concentration (Pb, Cd, Cr, Fe and Ni) in vegetables (mg/kg fw) were presented in Table 1. The concentration of heavy metals was increasing in the following order of Fe < Cd < Pb < Ni < Cr. The mean concentration of metals in vegetables was decreasing in order of pointed gourd > yard-long bean > cauliflower > teasel gourd > egg plant > ladies finger > taro > bitter gourd > Chinese okra > snake gourd > carrot > pumpkin > tomato > papaya > potato. Highest concentration of Cr was observed in vegetables and in Bangladesh, the sources of Cr are utilization of industrial and untreated water in vegetable production, use of chemical fertilizer and pesticides^14,30^. Compared with other vegetable types, the concentrations of Pb, Cr, Fe and Ni were higher in pod vegetables. Although studies found that leafy vegetables have high accumulation of heavy metals as they absorb large amount of heavy metals from soil and have large surface areas^31^. The concentration of heavy metals in different types of vegetables depends on the soil properties and accumulation capacities of vegetables^32^. The concentration of metals in vegetables were higher than the recommended value set by FAO/WHO except Fe. In the case of Fe, it is a naturally occurring element that is essential for plant growth and development. Plants have evolved mechanisms to regulate Fe uptake and distribution within their tissues to meet their physiological requirements while avoiding toxicity^33^. The highest concentration of Pb was found in cauliflower and yard-long bean. The mean concentration of Pb, Cd, Cr and Ni were higher than the previously studied literature in Bangladesh and Pakistan^4,7,34–36^ (Table 2) whereas the mean concentration of Fe in vegetables was higher than Ahmed et al.^34,36^ and similar to Sultana et al.^37^ (Table 2). Iron is an essential metal to prevent anemia however, excess consumption of iron may cause heart disease and liver damage^38^.

**Table 1:** Concentration of heavy metals (mg/kg fw) in commonly consumed vegetables in Bangladesh.

Common name	Scientific name	Type of vegetable	Heavy metals (mg/kg fw)				
			Pb	Cd	Cr	Fe	Ni
Bitter gourd (n = 9)	*Momordica charantia*	Fruit	1.696 ± 0.20	0.529 ± 0.23	14.047 ± 0.78	73.6 ± 0.43	4.4113 ± 0.15
Egg plant (n = 9)	*Solanum melongena L*	Fruit	5.603 ± 0.54	0.625 ± 0.23	13.347 ± 0.48	90.78 ± 0.42	5.63 ± 0.16
Snake gourd (n = 9)	*Trichosanthes cucumerina*	Fruit	1.982 ± 0.49	1.053 ± 0.10	12.178 ± 0.18	85.78 ± 2.4	4.650 ± 0.08
Pumpkin (n = 9)	*Cucurbita moschata*	Fruit	4.230 ± 0.29	0.402 ± 0.15	5.678 ± 0.20	61.83 ± 10.9	4.096 ± 0.002
Papaya (n = 9)	*Carica papaya*	Fruit	1.148 ± 0.31	0.042 ± 0.02	4.969 ± 0.17	79.21 ± 12.04	2.504 ± 0.10
Chinese okra (n = 9)	*Luffa acutangular*	Fruit	3.632 ± 0.87	0.321 ± 0.15	6.935 ± 0.07	95.07 ± 1.27	9.494 ± 0.06
Tomato (n = 9)	*Solanum lycopersicum*	Fruit	1.773 ± 0.65	0.925 ± 0.06	5.424 ± 0.04	83.14 ± 0.95	3.456 ± 0.15
Pointed gourd (n = 9)	*Trichosanthes dioica*	Pod	8.903 ± 0.38	0.419 ± 0.15	13.735 ± 0.25	124.65 ± 1.87	5.373 ± 0.22
Teasel gourd (n = 9)	*Momordica dioica*	Pod	3.888 ± 0.54	0.252 ± 0.09	12.767 ± 0.28	113.19 ± 2.28	8.006 ± 0.02
Ladies finger (n = 9)	*Abelmoschus esculentus*	Pod	4.472 ± 0.85	0.968 ± 0.15	12.592 ± 0.41	75.88 ± 0.57	4.996 ± 0.11
Yard-long Bean (n = 9)	*Vigna nguiculate ssp. Sesquipedalis*	Pod	12.593 ± 0.99	0.243 ± 0.11	9.367 ± 0.11	79.21 ± 5.35	6.063 ± 0.09
Carrot (n = 9)	*Daucus carota*	Root	1.785 ± 0.54	0.254 ± 0.10	8.623 ± 0.15	64.57 ± 10.61	4.272 ± 0.02
Taro (n = 9)	*Colocasia esculenta*	Root	7.746 ± 0.50	2.558 ± 0.20	8.115 ± 0.14	108.04 ± 1.03	3.632 ± 0.06
Potato (n = 9)	*Solanum tuberosum*	Root	0.298 ± 0.07	0.452 ± 0.10	3.912 ± 0.14	33.23 ± 1.104	3.055 ± 0.02
Cauliflower (n = 9)	*Brassica oleracea botrytis*	Stem	12.16 ± 0.78	1.724 ± 0.18	6.447 ± 0.05	92.65 ± 1.27	6.06 ± 0.08
Permissible limit			0.3^44^	0.05^45^	2.3^44^	450^46^	2.7^44^
% of vegetables exceeded permissible limit			93.3	93.3	100	0	93.3

**Table 2:** Comparison of average heavy metals (mg/kg fw) in vegetables with previous studies in Bangladesh.

Place of Study	Pb	Cd	Cr	Fe	Ni
Bangladesh (Noakhali) (Present study)	4.74	0.71	9.27	83.58	5.06
Bangladesh (Patuakhali)^35^	0.71	0.17	1.2		2.6
Bangladesh (Noakhali)^4^	3.7	0.058	0.64		1.44
Bangladesh (Dhaka)^7^	0.84	0.15	0.69		3.2
Bangladesh (Patuakhali)^15^	0.5	0.1	0.8		1.9
Bangladesh (Dhaka)^34^	3.9	0.62	1.7	65.95	3.0
Pakistan (Kasur)^36^	2.7	0.51	2.48	103	2.14
Pakistan (Lahore)^47^	0.3	0.25	1.29		3.2

### Principal component analysis and cluster analysis

**Figure 2:**
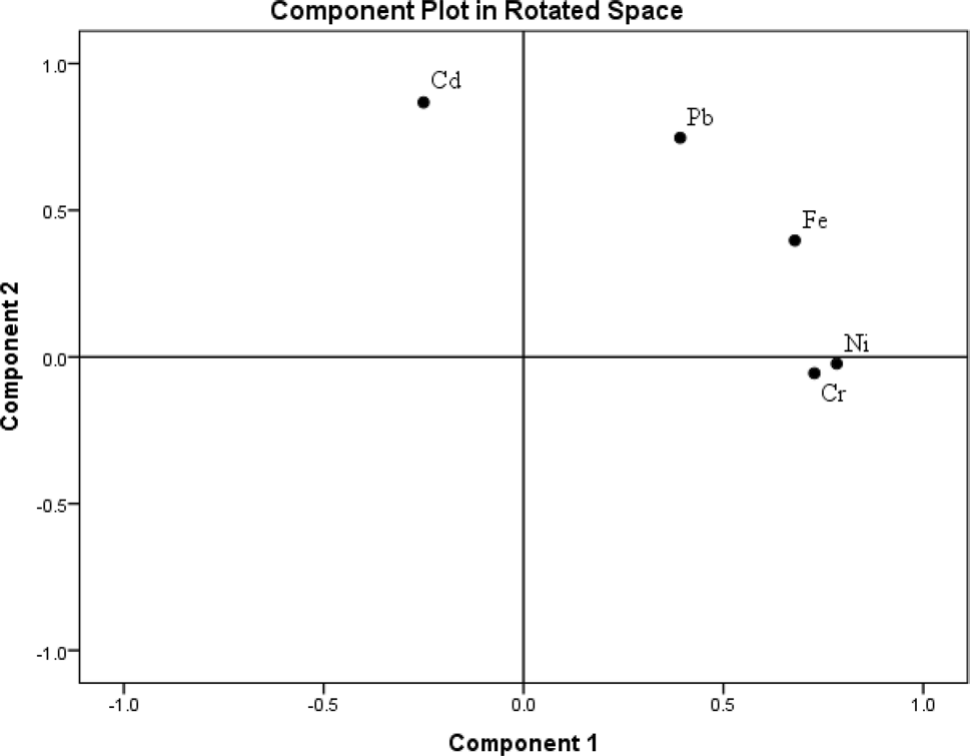
Principal component analysis of heavy metals in vegetables by varimax normalized rotation method showing loading of metals.

**Figure 3:**
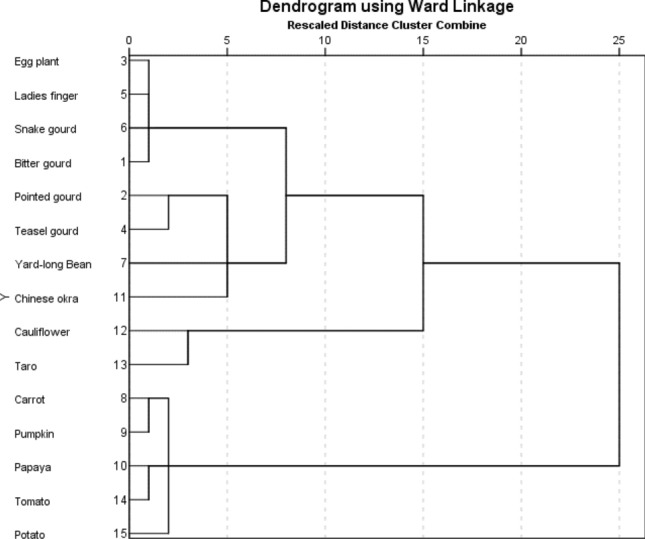
Cluster analysis (CA) of the vegetables from Noakhali region of Bangladesh.

Varimax-normalized rotation method was used for principal component analysis for estimating the factor loadings in each metal (Fig. 2). According to the results, two eigen values were greater than one and the first two component were explained the variance by 39.63% and 26.21% (Supplementary Table 4). PC1 revealed highest loadings for Pb, Cr, Ni and Fe which indicated that their source of origin was same and mostly contributed by anthropogenic activities such as use of fertilizers, pesticides, organic matters etc.^39,40^. Further, we used cluster analysis (CA) using Ward’s method with dendrogram for dividing the vegetables into different species (Fig. 3). Mean concentration of heavy metals was used for CA. Different cluster was formed between the vegetable species according to their similarity of nature.

### Dietary intake of heavy metals

Assessment of dietary intake of food is an essential tool for measuring the amount of nutrients intake which may be lead to deficiencies or health risks^41^. According to the average concentration of heavy metals in vegetable, dietary intake of metals for adult individual was estimated and presented in Table 3. Total dietary intake of metal of Pb, Cd, Cr, Fe and Ni from vegetables were 0.185, 0.0255, 0.3823, 3.4059, and 0.2057 mg/day for adults respectively. Dietary consumption of all of the metals except Cr from vegetables were less than the value of maximum tolerable dietary intake (MTDI) set by FAO/WHO which indicates that there is no potential human risk prior to consumption of vegetables from the studied area. This study showed that the contributions of studied vegetables to daily intake of Pb, Cd, Cr, Fe and Ni were 88%, 55%, 190%, 20% and 70% of the maximum tolerable dietary intake.

**Table 3:** Comparison of estimated daily intake (EDI) of heavy metals from vegetable with maximum tolerable daily intake (MTDI).

Vegetable type	Consumption rate (g/day/person)	EDI of heavy metals (mg/kg-weight/day)				
		Pb	Cd	Cr	Fe	Ni
**Fruits**						
Bitter gourd	191	0.0053	0.0014	0.0459	0.2334	0.0140
Egg plant	191	0.0160	0.0016	0.0423	0.2887	0.0179
Snake gourd	191	0.0061	0.0033	0.0388	0.2658	0.0150
Pumpkin	191	0.0125	0.0012	0.0187	0.1768	0.0130
Papaya	191	0.0033	0.0001	0.0153	0.2303	0.0082
Chinese okra	191	0.0112	0.0011	0.0223	0.3096	0.0301
Tomato	130	0.0026	0.0020	0.0116	0.1781	0.0077
EDI from fruits vegetables		0.057	0.0107	0.1949	1.6827	0.1059
**Pod**						
Pointed gourd	191	0.0294	0.0014	0.0439	0.3899	0.0173
Teasel gourd	191	0.0118	0.0007	0.0409	0.3684	0.0255
Ladies finger	130	0.0103	0.0019	0.0274	0.1653	0.0105
Yard-long Bean	130	0.0277	0.0005	0.0203	0.1780	0.0128
EDI from pod vegetables		0.0792	0.0045	0.1325	1.1016	0.0661
**Root and stem**						
Carrot	130	0.0038	0.0005	0.0187	0.1518	0.0093
Taro	130	0.0178	0.0055	0.0178	0.2328	0.0076
Potato	70.3	0.0004	0.0005	0.0046	0.0383	0.0035
Cauliflower	130	0.0268	0.0038	0.0138	0.1987	0.0133
EDI from root and stem vegetables		0.0488	0.0103	0.0549	0.6216	0.0337
Total intake from vegetables		0.185	0.0255	0.3823	3.4059	0.2057
MTDI		0.21^44^	0.046^44^	0.2^48^	17^44^	0.3^49^

### Non-carcinogenic and carcinogenic health risk

The non-carcinogenic risk in terms of target hazard quotient (THQ), total target hazard quotient (TTHQ) and hazard index (HI) and carcinogenic risk of consuming studied vegetables were presented in Table 4. The THQ values of Pb and Cd for almost all species of vegetable were higher than 1, indicated that consumption of these vegetables might cause non-carcinogenic risk. The THQ values of the studied vegetables (except Cr) were higher than 1 which indicated that people might have potential health risk prior to vegetables consumption. A study in India revealed potential health effects from Cd and Ni due to the value of HQ exceeded the safe limit^42^. The ranking order of total THQ for vegetables species were cauli flower > taro > pointed gourd > yard-long bean > eggplant > Chinese okra > snake gourd > teasel gourd > pumpkin > ladies finger > bitter gourd > tomato > carrot > papaya > potato. Considering all the metals, the total THQ (sum of individual metals or HI) was 35.36, 32.44, and 27.017 for fruit vegetable, pod vegetable and root and stem vegetable respectively, in a total 94.797 which is > 1 indicating that these vegetables were not safe for human consumption and consumption on regular basis is not recommended. It is stated that exposure to two or more heavy metals can cause additive or interactive effects^24^.

**Table 4:** Carcinogenic and non-carcinogenic risk of heavy metals consuming vegetables in Bangladesh.

Vegetable type	Target Hazard Quotient (THQ)					TTHQ	Total Cancer risk (TCR)		
	Pb	Cd	Cr	Fe	Ni		Pb	Cd	Cr
**Fruits**									
Bitter gourd	1.5352	1.4301	0.0306	0.3335	0.7044	4.0338	4.6E−05	0.02145	0.01931
Egg plant	4.584	1.6895	0.0282	0.4124	0.8992	7.6133	1.0E−04	0.02534	0.01777
Snake gourd	1.7499	3.3729	0.0259	0.3797	0.7535	6.2819	5.2E−05	0.05059	0.01632
Pumpkin	3.5748	1.2672	0.0124	0.2526	0.6516	5.7586	1.0E−04	0.01901	0.00786
Papaya	0.9554	0.1546	0.0102	0.3291	0.4146	1.8639	2.8E−05	0.00232	0.00643
Chinese okra	3.2279	1.1365	0.0149	0.4423	1.5065	6.3281	9.6E−05	0.01705	0.00939
Tomato	0.7552	2.0725	0.0077	0.2544	0.3896	3.4794	2.3E−05	0.03108	0.00489
TTHQ from fruit vegetables	16.3824	11.1233	0.1299	2.404	5.3194	HI = 35.36			
**Pod**									
Pointed gourd	8.4221	1.4269	0.0292	0.557	0.8658	11.301	2.0E−04	0.02141	0.01845
Teasel gourd	3.3793	0.795	0.0273	0.5263	1.2757	6.0036	1.0E−04	0.01193	0.01721
Ladies finger	2.9689	1.9585	0.0183	0.2362	0.5292	5.7111	8.8E−05	0.02937	0.01155
Yard-long Bean	7.9423	0.5649	0.0135	0.2543	0.6445	9.4195	2.0E−04	0.00847	0.00853
TTHQ from pod vegetables	22.7126	4.7453	0.0883	1.5738	3.3152	HI = 32.44			
**Root and stem**									
Carrot	1.0935	0.5487	0.0125	0.2169	0.4661	2.3377	3.3E−05	0.00823	0.00789
Taro	5.0885	5.5826	0.0118	0.3326	0.3848	11.4003	1.0E−04	0.08374	0.00748
Potato	0.127	0.523	0.003	.00054	0.17	0.831	3.8E−06	0.00785	0.00195
Cauliflower	7.67	3.8193	0.0092	0.2839	0.6662	12.4486	2.0E−04	0.05729	0.00581
TTHQ from root and stem vegetables	13.97	10.47	0.0365	0.8339	1.68	HI = 27.017			
TTHQ	53.065	26.3386	0.2547	4.8112	10.3146	94.797	1.4E−03	0.39514	0.16084

**Figure 4:**
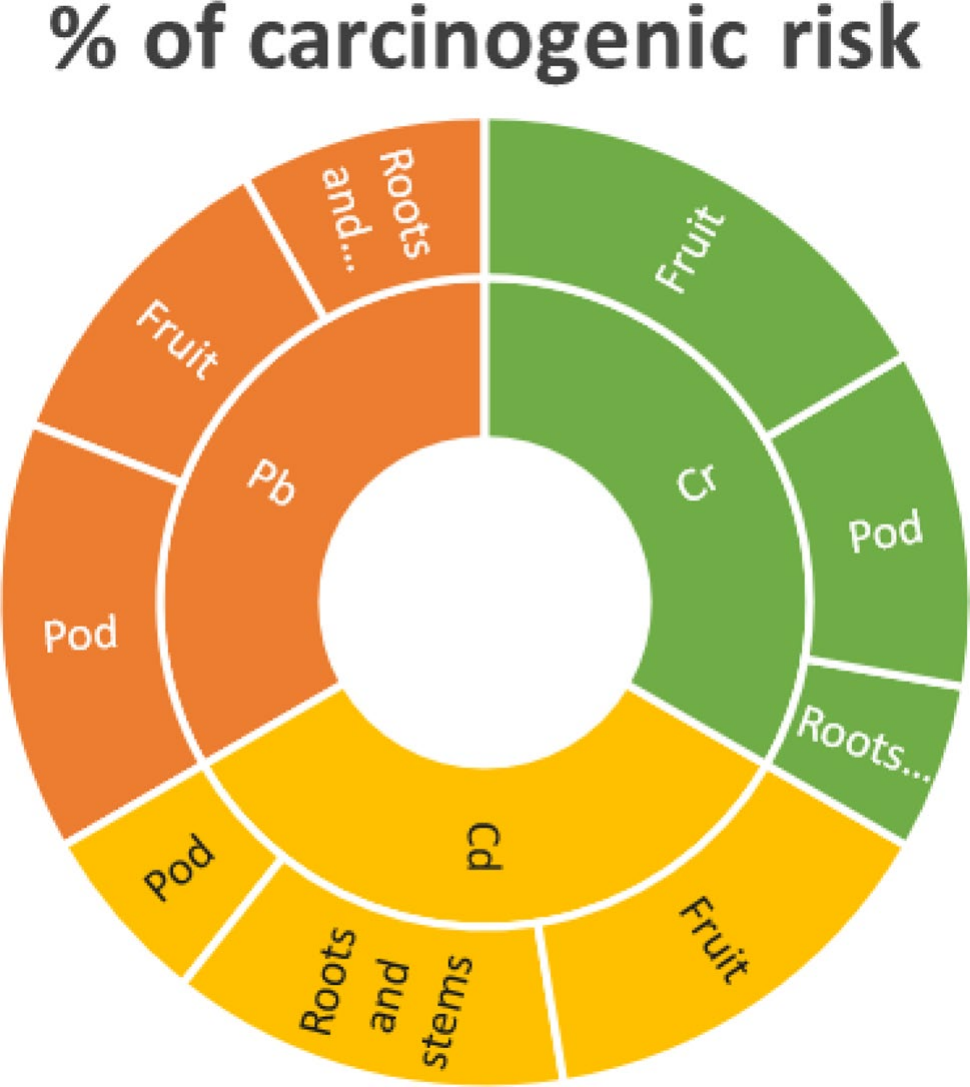
Cumulative cancer risk (%) of heavy metals according to different studied vegetable types.

As Pb, Cd and Cr contributed both non-carcinogenic and carcinogenic health risk depending on the exposure dose and duration, the present study estimated the TCR values of Pb, Cd and Cr due to exposure from several vegetables presented in Table 4. TCR values for Pb ranged from 3.8E-06 to 2.0E-04; for Cd, TCR ranged from 2.3E−03 to 8.3E−02 whereas 1.95E−03 to 1.93E−02 for Cr in vegetables. The TCR values of Pb for pointed gourd, yard-long bean and cauliflower were higher than 1.0E−04^29^ which indicated that potential carcinogenic risk of the consumers in the studied area. TCR values of Pb for other vegetable except potato were lying between the range of 1.0E-06 to 1.0E-04 which are considered as acceptable^29^. The TCR of Cd and Cr from the consumption of vegetables were higher than USEPA acceptable limit risk (10^–4^) indicating that consumers of these vegetables are exposed to Cd and Cr with a life-time cancer risk. The percentage of carcinogenic risk of Cr (49.61%) and Cd (42.22%) were higher among consumers who intake fruit vegetables whereas higher carcinogenic risk for Pb was found in pod vegetables (42.93%) consumption (Fig. 4). The cumulative cancer risk percentage of Pb (24.6%) and Cr (16.66%) from roots and stems consumption were lower.

In the present study, we have several limitations. We didn’t measure the accumulation rate of each vegetable from soil to edible parts. We didn’t account the other sources of heavy metal exposure such as dermal, nasal which may associate with health risk to humans. The effect of environment such as seasonal variations on heavy metals concentration in vegetables also need to be addressed. Given the well-established presence of arsenic contamination in Bangladesh^43^ and its potential impact on agricultural produce^43^, the omission of arsenic analysis in this study is a significant shortcoming. Limited resources and analytical capabilities for arsenic detection contributed to the exclusion of arsenic analysis in this study as arsenic analysis in vegetables typically requires hydride generation system and the additional sample preparation steps involved in arsenic analysis compared to the detection of heavy metals like lead, cadmium, chromium, nickel, and iron^43^. Another limitation is that the AAS technique used in this study measures the total chromium content, which includes both Cr (III) and Cr (VI), without distinguishing between the different oxidation states. In the absence of speciation data, applying the CPSo value for Cr (VI) to total chromium concentrations may overestimate the carcinogenic risk, as it assumes that all the chromium present in the samples is in the hexavalent form.

## Conclusion

The present study revealed the concentrations of heavy metals in commonly consumed vegetables in Noakhali district of Bangladesh and assessed the potential health risk prior to vegetable consumption in terms of THQ and TCR. In Noakhali, maximum of vegetables had toxic metals (Pb, Cd, Cr and Ni) which are higher than the maximum allowable concentration (MAC) however, in case of EDI, only Cr exceed the permissible limit (MTDI) set by FAO/WHO. The total THQ for Pb, Cd, Fe and Ni were > 1 through vegetables consumption indicating potential health risk. Consumption of fruit vegetables, pod vegetables and root and stem vegetables would be unsafe as HI value was > 1. Considering TCR values, the total TCR values for Pb, Cd and Cr were higher than 1.0E−04 suggesting potential health risk from vegetable consumption. The findings emphasize the need for immediate action to mitigate these risks and ensure food safety. Future research should focus on identifying the sources of contamination, assessing other exposure routes, and exploring the influence of environmental factors. Policymakers and stakeholders must collaborate to develop and implement stringent regulations, monitoring programs, and remediation techniques to address this pressing issue. Public awareness campaigns are crucial to educate farmers and consumers about the risks and promote safe agricultural practices.

### Supplementary Information

Supplementary Information.

## Data Availability

The datasets generated and/or analyzed during the current study are available from the corresponding author on reasonable request.
