# Supplementary material for: Human health risk assessment of heavy metals in vegetables of Bangladesh
Source: Sci Rep. 2024 Jul 6;14:15616. doi: 10.1038/s41598-024-65734-6 (PMC11227495; doi:10.1038/s41598-024-65734-6)
Supplement: Supplementary file 1 — Supplementary Information. [file 41598_2024_65734_MOESM1_ESM.pdf]

Human health risk assessment of heavy metals in vegetables of Bangladesh

Akibul Islam Chowdhury[1,2], Lincon Chandra Shill[1], M Maruf Raihan[1], Rumana Rashid[3], Md. Nizamul Hoque Buiyan[4], Sompa Reza[4], Mohammad Rahanur Alam[1,5,*]

[1]Department of Food Technology and Nutrition Science, Noakhali Science and Technology University, Noakhali, Bangladesh

[2]Department of Nutrition and Food Engineering, Daffodil International University, Savar, Dhaka, Bangladesh.

[3]Department of Public Health Nutrition, Primeasia University, Dhaka, Bangladesh.

[4]Institute of Nutrition and Food Science, University of Dhaka, Dhaka, Bangladesh.

[5]Department of Nutrition, University of Tennessee, Knoxville, Tennessee, USA

Table S1: Analytical conditions for measurement for trace elements in aqueous solution using AAS

Elements	Lamp current (mA)	Wave length (nm)	Slit (nm)	Mode	LOD (µg/L)
Cr	7.0	357.87	0.7	GFAA	0.004
Fe	10.0	248.33	0.2	FAA	5
Cd	8.0	228.80	0.7	GFAA	0.002
Pb	8.0	283.30	0.7	GFAA	0.05
Ni	7.0	232.0	0.2	GFAA	0.07

Table S2: Percentage of recovery of toxic metals

Elements	Calibration curve (R^2)	Amount used for spiking (µg/g)	Amount recovered (µg/g)	Percentage of recovery (% R)
Cr	0.997705	20	19.43	97.15
Fe	0.997020	20	18.87	94.35
Cd	0.998202	20	20.31	101.55
Pb	0.995125	20	19.54	97.70
Ni	0.996534	20	18.34	91.70

Table S3: Data required for estimating EDI, THQ and CR

Parameters		Reference
MC (metal concentration) in mg/kg fw		Present study
IR (Ingestion rate) in g/day/person		[1]
BW (body weight)	60 kg	[2]
ED (exposure duration)	70 years	[3]
AT (average exposure time)	25550 days	[3]
RfD	Cd, Cr, Ni, Pb, Fe	[4]
CPSo	Pb, Cd, Cr	[5]

Table S4: Explanation of total variance and component matrix for the heavy metals in vegetables

Total Variance Explained

Component	Initial Eigenvalues			Extraction Sums of Squared Loadings			Rotation Sums of Squared Loadings		
	Total	% of Variance	Cumulative %	Total	% of Variance	Cumulative %	Total	% of Variance	Cumulative %
1	1.982	39.632	39.632	1.982	39.632	39.632	1.820	36.399	36.399
2	1.311	26.211	65.843	1.311	26.211	65.843	1.472	29.445	65.843
3	.755	15.097	80.940						
4	.540	10.801	91.741						
5	.413	8.259	100.000						
	Component Matrix					Rotated component matrix			
	PC1	PC2				PC1	PC2		
Pb	0.708	0.458				0.392	0.747		
Cd	0.208	0.879				-0.250	0.868		
Cr	0.607	-0.405				0.728	-0.055		
Fe	0.786	0.013				0.679	0.397		
Ni	0.671	-0.404				0.783	-0.023		

Extraction Method: Principal Component Analysis.

References:

1 HIES. Preliminary Report on Household Income and Expenditure Survey-2010. . (Bangladesh Bureau of Statistics, Statistics Division, Ministry of Planning, Dhaka, Bangladesh., 2011).

2 EPA, U. Integrated Risk Information System-Database (IRIS). (2007).

3 USEPA. Regional Screening Level (RSL) Summary Table: November 2011. (2011).

4 EPA, U. Risk-Based Concentration Table: Technical Back-Ground Information. *Environmental Protection Agency, Washington, DC* (2006).

5 OHHEA. Appendix A: Hot Spots Unit Risk and Cancer Potency Values. (2023).
